# Compositional Variation in FAPb_1–*x*_Sn_*x*_I_3_ and Its Impact on the Electronic Structure: A Combined Density Functional Theory and Experimental Study

**DOI:** 10.1021/acsami.2c00889

**Published:** 2022-05-05

**Authors:** Simon Kahmann, Zehua Chen, Oleh Hordiichuk, Olga Nazarenko, Shuyan Shao, Maksym V. Kovalenko, Graeme R. Blake, Shuxia Tao, Maria A. Loi

**Affiliations:** † Photophysics and OptoElectronics Group, Zernike Institute of Advanced Materials, University of Groningen, Nijenborgh 4, NL-9747 AG Groningen, The Netherlands; ¶ Materials Simulation and Modelling, Department of Applied Physics, Eindhoven University of Technology, 5600 MB Eindhoven, The Netherlands; § Center for Computational Energy Research, Department of Applied Physics, Eindhoven University of Technology, 5600 MB Eindhoven, The Netherlands; ∥ Department of Chemistry and Applied Biosciences, ETH Zürich, Vladimir Prelog Weg 1, CH-8093 Zürich, Switzerland; ⊥ EMPA-Swiss Federal Laboratories for Materials Science and Technology, Überlandstraße 129, CH-8600 Dübendorf, Switzerland; ○ Solid State Materials for Electronics, Zernike Institute of Advanced Materials, University of Groningen, Nijenborgh 4, NL-9747 AG Groningen, The Netherlands

**Keywords:** lead−tin mixed perovskites, single crystals, crystallography, band bending, DFT calculations, photoluminescence

## Abstract

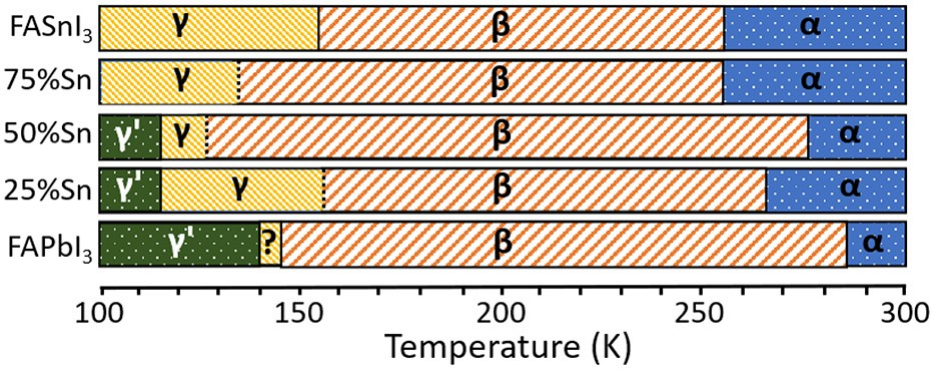

Given their comparatively narrow band gap, mixed Pb–Sn iodide perovskites are interesting candidates for bottom cells in all-perovskite tandems or single junction solar cells, and their luminescence around 900 nm offers great potential for near-infrared optoelectronics. Here, we investigate mixed FAPb_1–*x*_Sn_*x*_I_3_ offering the first accurate determination of the crystal structure over a temperature range from 293 to 100 K. We demonstrate that all compositions exhibit a cubic structure at room temperature and undergo at least two transitions to lower symmetry tetragonal phases upon cooling. Using density functional theory (DFT) calculations based on these structures, we subsequently reveal that the main impact on the band gap bowing is the different energy of the s and p orbital levels derived from Pb and Sn. In addition, this energy mismatch results in strongly composition-dependent luminescence characteristics. Whereas neat and Sn-rich compounds exhibit bright and narrow emission with a clean band gap, Sn-poor compounds intrinsically suffer from increased carrier recombination mediated by in-gap states, as evidenced by the appearance of pronounced low-energy photoluminescence upon cooling. This study is the first to link experimentally determined structures of FAPb_1–*x*_Sn_*x*_I_3_ with the electronic properties, and we demonstrate that optoelectronic applications based on Pb–Sn iodide compounds should employ Sn-rich compositions.

## Introduction

Lead–tin mixed halide perovskites are an intriguing class of materials because of their narrower band gap compared to their neat parent compounds.^1^ The smaller band gap energy renders them interesting for single junction solar cells^2−4^ but particularly so for bottom cells in all-perovskite tandem devices.^5−7^ Simultaneously, the relative reduction of the lead content is often considered beneficial from an environmental point of view, and mixed compounds exhibit a higher stability than neat tin-based perovskites, which are prone to degradation.^8,9^

Whereas a wide field of reports on applications and pathways for performance improvements of these mixed Pb–Sn compounds has emerged, there remains a paucity of fundamental insights. In the absence of experimental data, for example, computational studies commonly rely on calculated crystal structures. Moreover, neat Pb- or Sn-based compounds exhibit several crystallographic phase transitions upon temperature variation,^10−13^ and in the absence of experimental data, it is often tacitly assumed that mixed Pb–Sn compounds behaved analogously.

One of the intriguing aspects of mixed lead–tin halide perovskites is the pronounced bowing of the band gap.^1^ Over a broad compositional range, their gap is narrower than for either end compound. Such bowing can generally occur due to three different sources:^14^ (i) changes in the volume deformation potential, i.e., unit cell compression or dilation with respect to the neat compounds, (ii) chemical effects, i.e., the intermixing of different atomic orbitals leading to different band energies, (iii) structural relaxation effects, for example, due to lattice distortions or octahedral rotation.

Several reports have addressed this issue for mixed lead–tin compounds with varying conclusions. For MAPb_1–*x*_Sn_*x*_I_3_, Im et al. argued that the competition between the spin–orbit coupling (SOC) and the lattice distortion was responsible.^150^ In contrast, Eperon et al. claimed that the SOC did not affect the band gap bowing and proposed that a short-range ordering of Pb and Sn atoms contributed to the nonlinearity.^5^ Goyal et al. subsequently proposed that it was primarily a consequence of chemical effects, namely, the mismatch in energy between s and p atomic orbitals of Pb and Sn.^15^ The authors of the latter study furthermore suggested that SOC, structural distortions, and short-range ordering all had a negligible impact on the band gap bowing. Similar work on CsPb_1–*x*_Sn_*x*_I_3_ was performed by Valadares et al., who noted the same impact of the orbital energies but also held that spin–orbit interactions were important.^16^ Notably, in the absence of the required high-quality single crystals, few of these studies considered actually determined crystal structures despite the large effect the structure has on the calculated bowing.^17^

Neat metal halide perovskites exhibit a specific defect chemistry.^18,19^ Whereas Sn-based compounds are generally prone to the formation of Sn vacancies and easy self-oxidation, the impact on Pb-based compounds generally stems from halide interstitials or A-site species. Compositional variation will consequently vary the presence and role of these defects, but few reports are currently available on their impact.^20^

Here, we synthesized single crystals of FAPb_1–*x*_Sn_*x*_I_3_ (*x* = 0.25, 0.5, and 0.75) that allowed for precise determination of their crystal structure over a broad temperature range. All compounds exhibit a cubic structure at room temperature (α-phase) and undergo at least two transitions upon cooling (β- and γ-phase). On the basis of these structures, we performed density functional theory (DFT) calculations to address the controversy around the band gap bowing observed in the spectroscopic experiments. Importantly, this is the first study on FAPb_1–*x*_Sn_*x*_I_3_ that bases computational data on experimentally determined structures, thereby assuring maximum accuracy. Photoluminescence (PL) spectroscopy reveals that compositions of low Sn content are particularly affected by defect states, which reduce the band edge luminescence at room temperature and manifest in an increasingly bright low-energy emission upon cooling. Considering the results from the DFT calculations, we link the composition-dependent impact of defects to the chemical origin of the conduction and valence band edges, namely, the Pb- and Sn-derived states of the conduction and valence band, respectively. This forms a stark contrast to the wide-held idea that Sn is the source of defectiveness and degradation in perovskites, whereas high Pb content entails higher stability and better performance.

## Experimental Section

### Single Crystal Synthesis

#### 75% Sn

FAOAc + SnO (+PbI_2_) in HI (57%)/H_3_PO_2_ (50%) (1:1 by volume) (1.2 molar excess of FAOAc) was used. The HI/H_3_PO_2_ mixture was degassed by slow bubbling of Ar for 15–20 min. The solutions were prepared by dissolving powders in the mixture of acids at 80–85 °C. The obtained solution was distributed over two 15 mL polypropylene centrifuge tubes (using polytetrafluoroethylene cannulas), which were subsequently placed into an incubator at 70 °C and then cooled to 25 °C. The cooling rate was 0.1 °C per 12 min.

Formed crystals were washed with the following procedure: First, the mother liquor was removed by a cannula; next, the crystals were washed twice with the mixture of HI (57%) and H_3_PO_2_ (50%) (1:1 by volume). The mixture was previously degassed by five freeze–pump–thaw cycles and cooled to −20 to −35 °C; subsequently, the crystals were washed twice with anhydrous ethanol cooled to −20 °C and finally dried under the flow of argon at 60 °C overnight.

#### 50% Sn

First, 0.178 g of Sn (1.5 mmol) was dissolved in a degassed solution of 3 mL of HI and 5 mL of H_3_PO_2_ upon heating; next, 0.692 g of PbI_2_ (1.5 mmol) and 0.37 g (3.55 mmol) of FAOAc were added. A black precipitate formed, which further dissolved upon heating to 100–115 °C to form a yellow transparent solution. The stirring was discontinued, and the temperature of the glycerol bath was set to 70 °C.

Overnight, a few small crystallites grew (about or a bit smaller than 1 mM). On day 2, the temperature was reduced by 10 °C over two steps, and on day 3, the temperature was further reduced to 35 °C until the end of the day.

On day 4, the day crystals were filtered under a N_2_ flow and dried on a sand bath at 70–80 °C under vacuum. Some yellow precipitate formed on the crystal surface upon drying. The crystals were subsequently transferred into a glove box and rinsed with DMF and dried. No further precipitate formed.

#### 25% Sn

0.3 mmol of FAOAc, 0.75 mmol of Sn, and 2.25 mmol of PbI_2_ were used for the synthesis in a solution of 3 mL of HI and 6 mL of H_3_PO_2_.

Sn was first dissolved upon heating in HI, and next FAOAc, PbI_2_, and degassed H_3_PO_2_ (under the N_2_ stream) were added. All the components and a formed black precipitate dissolved upon heating to form a yellow transparent solution. The temperature was reduced to 80–85 °C within about 3 h and further decreased to about 40–45 °C over the course of 10 days. The solution was filtered warm (40–45 °C) since at RT yellow-phase FAPbI_3_ crystallized out of solution. Upon drying under vacuum, some yellow precipitate formed on the crystals, which was rinsed away with DMF (under a N_2_ atmosphere).

#### Chemicals and Reagents

Lead(II) iodide (PbI_2_, 99%) was purchased from Sigma-Aldrich. Tin(II) oxide (SnO, 99%), formamidine acetate (FAOAc, 99%), and hydriodic acid (57% aqueous solution, stabilized with 1.5% hypophosphorous acid) were purchased from ABCR. Hypophosphorous acid (50% solution in water) and anhydrous ethanol (99,8%) were purchased from Acros Organics.

### Thin Film Preparation

The chemical reagents for thin film samples were used as received. FAI (>98%) and PbI_2_ (>99.99%) were purchased from TCI EUROPE N.V. SnI_2_ (99.99%), SnF2 (>99%), DMF, and DMSO (99.8%) were purchased from Sigma-Aldrich.

The FAPbI_3_ solution was made by dissolving 1 mM FAI and 1 mM PbI_2_ in 1 mL of mixed DMF and DMSO (4:1 volume ratio). FASnI_3_ was made by dissolving 1 mM FAI, 1 mM SnI_2_, and 0.1 mM SnF_2_ in 1 mL of mixed solvents of DMF and DMSO (4:1 volume ratio). Then, FAPb_1–*x*_Sn_*x*_I_3_ solutions were made by mixing FAPbI_3_ and FASnI_3_ solutions with a volume ratio of (1 – *x*)/*x*.

Quartz substrates were cleaned using an ultrasonication bath in soapy water and rinsed sequentially with deionized water, acetone, and isopropyl alcohol. The substrates were dried at 140 °C for 20 min and then subjected to UV–ozone treatment for 20 min. The substrates were transferred into a nitrogen-filled glovebox. The FAPb_(1–*x*)_Sn_*x*_I_3_ films were spin-coated from the corresponding solutions at 4000 rpm for 60 s. Diethyl ether was used as the antisolvent 12 s after spinning commenced. Finally, the FAPb_(1–*x*)_Sn_*x*_I_3_ films were annealed at 100 °C for 10 min.

### Crystallography

Single crystal X-ray diffraction was performed using a Bruker D8 Venture diffractometer operating with Mo Kα radiation and equipped with a Triumph monochromator and a Photon100 area detector. A small piece of approximate dimensions of 0.1 mM was cut from a larger crystal in a nitrogen-filled glovebox and was mounted in a nylon loop using cryo-oil. The sample was then removed from the glovebox and quickly transferred to the diffractometer, where it was cooled using a flow of dry nitrogen using an Oxford Cryosystems Cryostream Plus. The data were processed using the Bruker Apex III software. The structure was solved and refined using the SHELXTL software.^21^

### Computational Methods

To model the mixed FAPb_1–*x*_Sn_*x*_I_3_ alloy, we start with the cubic unit cell of room-temperature FASnI_3_ determined in the previous XRD experiments.^13^ The structure parameters are given in Table 1. We then expand the cubic unit cell of FASnI_3_ to a 2 × 2 × 2 cubic supercell and replace the Sn by Pb to create 0%, 25%, 50%, 75%, and 100% Sn compounds, respectively. The total number of possible configurations is 128. For the cubic supercell with perfect O_*h*_ symmetry, the eight metal sites are equivalent, which reduces the total number of inequivalent configurations to 14. Here, the orientation of the FA cations for the starting configurations are treated to have the same direction, which is then slightly adjusted by following geometric relaxations. As we will show further on in this work, the calculated bowing parameter is close to the result obtained using a polymorphous crystal structure for both alloy and end-point compounds in the cubic phase, where the orientation of FA cations are disordered.^17^ This indicates the orientation of FA cations has a minor impact on the band gap bowing in mixed FAPb_1–*x*_Sn_*x*_I_3_.

**Table 1:** Crystal Structure Parameters for Pb:Sn Mixed Crystals between Room Temperature and 100 K^a^

	100% Sn	75% Sn	50% Sn	25% Sn	0% Sn
α	cubic	cubic	cubic	cubic	cubic
	*Pm3̅m*	*Pm3̅m*	*Pm3̅m*	*Pm3̅m*	*Pm3̅m*
	*a* = 6.3074(15)	*a* = 6.3158(18)	*a* = 6.344(7)	*a* = 6.3401(7)	*a* = 6.362
β	tetragonal	tetragonal	tetragonal	tetragonal	tetragonal
	*P4/mbm*	*P4/mbm*	*P4/mbm*	*P4/mbm*	*P4/mbm*
	*a* = *b* = 8.8822(6)	*a* = *b* = 8.8798(7)	*a* = *b* = 8.8904(4)	*a* = *b* = 8.9081(6)	*a* = *b* = 8.922
	*c* = 6.2698(6)	*c* = 6.2790(5)	*c* = 6.2877(3)	*c* = 6.3004(4)	*c* = 6.326
γ, γ′	tetragonal	tetragonal	tetragonal^b^	tetragonal^b^	tetragonal^c^
	*P4bm*	*P4/mbm*/*P4bm*	??	??	*P4/mbm*
	*a* = *b* = 8.8379(11)	*a* = *b* = 8.8195(6)	*a* = *b* = 8.783(10)	*a* = *b* = 8.842(7)	*a* = *b* = 8.875
	*c* = 12.4066(17)	*c* = 12.4572(9)	*c* = 6.191(6)	*c* = 6.238(6)	*c* = 6.279
			*q* = (0, 0, 0.1597(8))	*q* = (0, 0, 0.1731(10))	

a Parameters given for 298, 200, 100 K, as determined by single crystal X-ray diffraction; data on the two end compounds taken from.^12,13^ Dimensions of the unit cells are given in Å.

b Incommensurate.

c Conflicting reports.

The Special Quasirandom Structures (SQS) method^26^ is used to obtain the best approximation to an ideal infinite random distribution of Sn and Pb in the 4 × 4 × 4 supercells containing 64 formula units. To reduce the computational cost, we replace all the organic cations with inorganic cation Cs, since the A cation in ABX_3_ does not directly contribute to band edge states and the orientation of FA cations does not impair the band gap bowing as mentioned above. The SQS are generated with the ATAT code^27^ considering pairs and triplets with B site cation–cation correlation that gives the best match to the true disordered solid solution.

All calculations are performed within density functional theory (DFT). We use the projected augmented wave (PAW)^22^ method and the generalized gradient approximation (GGA)/Perdew–Burke–Ernzerhof (PBE)^23,24^ functional with and without spin–orbit coupling (SOC) as implemented in the Vienna ab initio simulation package (VASP).^25^ The plane-wave kinetic energy cutoff is set at 500 eV. We use 8 × 8 × 8, 4 × 4 × 4, and Γ-point k-point Brillouin zone samplings for the unit cell, 2 × 2 × 2, and 4 × 4 × 4 supercells, respectively. The lattice constant and shape of the inequivalent configurations at each discrete *x* are fixed to the corresponding cubic supercell of room-temperature experimental data in Table 1. The atomic positions for each possible configuration are fully relaxed. The energy and force convergence parameters are set at 0.01 meV and 0.01 eV/Å, respectively. The single point calculations including SOC are based on the equilibrium structures optimized by the PBE functional.

### Photoluminescence Spectroscopy

Without having been exposed to air, thin films were mounted into a cryostat (Oxford Optistat CF) working with both helium exchange gas and a coldfinger. Samples were kept for 15 min at every new temperature step prior to measurement. Excitation occurred at 3.1 eV (400 nm) using the second harmonic of a mode-locked Ti:sapphire laser (Mira 900, coherent) at a repetition rate of 76 MHz. Steady-state spectra were collected with an InGaAs detector from Andor (iDus 1.7 μm). The excitation beam was spatially limited by an iris and focused with a 150 mM focal length lens.

## Results and Discussion

We synthesized single crystals through a slow-cooling process used previously for neat FASnI_3_.^13^ A typical procedure yields two to ten black crystals with side lengths ranging from 0.5 to 3 mm.

**Figure 1:**
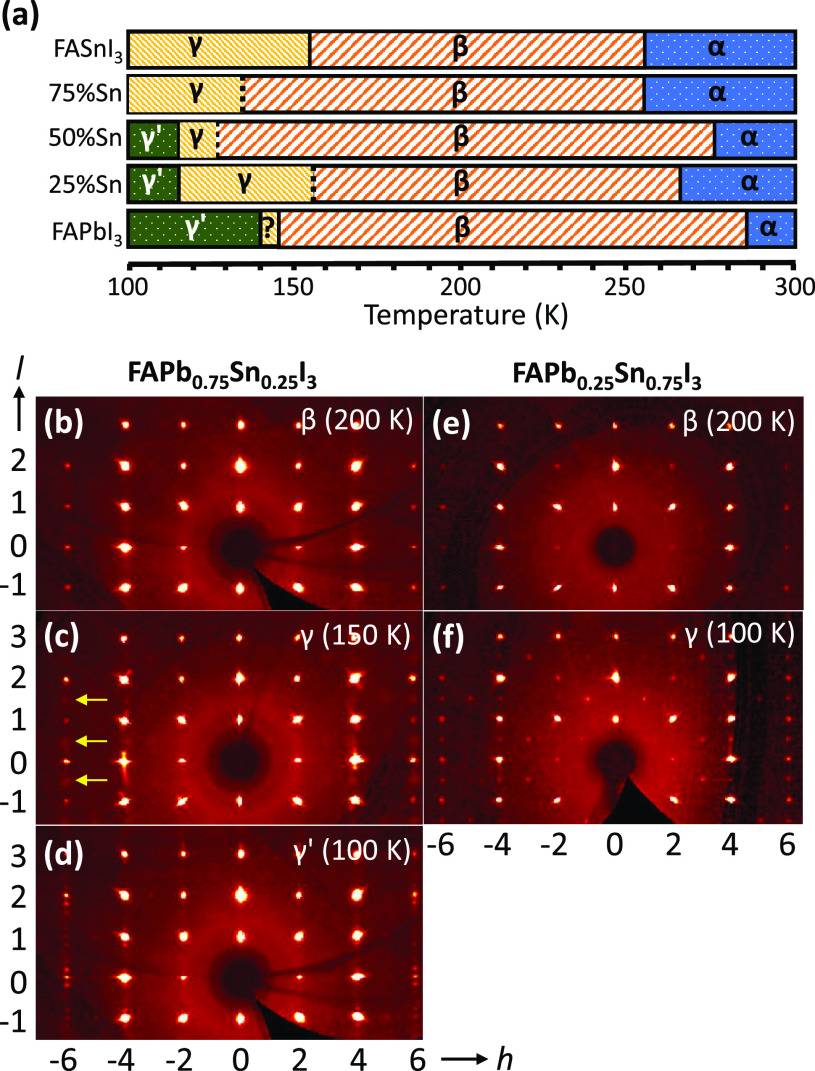
(a) Summary of phases exhibited by all samples studied as a function of temperature. Dotted lines indicate gradual or complex phase transitions. (b–f) (h0l) reciprocal lattice planes reconstructed from raw X-ray diffraction data collected on FAPb_0.75_Sn_0.25_I_3_ and FAPb_0.25_Sn_0.75_I_3_, showing the evolution of the diffraction patterns in the β-, γ-, and γ′-phases (indexing is referred to the tetragonal β-phase). The γ-phase of FAPb_0.25_Sn_0.75_I_3_ exhibits strong half-integer *l*-spots, whereas these are much weaker and more diffuse for FAPb_0.75_Sn_0.25_I_3_ (indicated with arrows). The γ′-phase of FAPb_0.75_Sn_0.25_I_3_ exhibits “satellite” spots in the *l*-direction, indicative of a modulated structure.

We performed single crystal X-ray diffraction (XRD) over the range from room temperature down to 100 K to determine the dependence of the crystal structure on the composition. We find that all compounds undergo two or more phase transitions over this temperature range (Figure 1a). Table 1 summarizes the crystallographic data along with the previously determined parameters for neat FASnI_3_^13^ and published data on FAPbI_3_ as reported by Weber et al.^12^Figure 1a shows the sequence of phase transitions undergone for each composition.

When Sn is replaced with Pb, the unit cell generally increases in size due to the larger radius of Pb^2+^ compared to Sn^2+^, whereas all compositions maintain cubic *Pm3̅m* symmetry at room temperature (see Table 1). This is referred to in the literature as the α-phase in which the FA cations are fully disordered with no preferred orientation. Upon cooling, all compounds first exhibit a phase transition to a tetragonal *P4/mbm* β-phase between 285 and 255 K (see the XRD pattern in Figure 1b,e) as previously reported for the end members of the series FASnI_3_^13^ and FAPbI_3_.^12^ As discussed before,^13^ this transition involves a doubling of the unit cell volume and the *a*- and *b*-axes being enlarged by a factor of . The Sn/PbI_6_ octahedra undergo a cooperative rotation around the *c*-axis, and the FA molecules become locked in an orientation perpendicular to the *ab*-plane; the central C atom lies on a mirror plane perpendicular to the *c*-axis, which implies that the orientation of the molecule is 2-fold disordered. This cubic–tetragonal transition always gives rise to the formation of three twin domains formed by 90° rotations around the tetragonal [110] and [11̅0] axes.

The nature of the second transition to the γ-phase strongly depends on the composition. We previously showed that FASnI_3_ undergoes a doubling of the *c*-axis below 155 K, which involves full orientational ordering of the FA molecules and also breaks inversion symmetry with the adoption of space group *P4bm*, while the twinning of the β-phase is retained.^13^ The 75%, 50%, and 25% Sn containing crystals exhibit a similar phase change involving *c*-axis doubling at ∼135, ∼125, and ∼155 K, respectively. The *c*-axis doubling is evidenced by the appearance of half-integer diffraction spots in the reciprocal *l*-direction (see Figure 1f). In contrast to the sharp transition from *P4/mbm* to *P4bm* in FASnI_3_, this change occurs more slowly for the mixed compositions with new diffraction spots appearing at the transition and slightly increasing in intensity as the temperature is lowered further (Figure 1b–d). The data for 75% Sn do not allow for a clear distinction between a centrosymmetric *P4/mbm* or a noncentrosymmetric *P4bm* phase to be made. The fit for the *P4bm* model is marginally better (R1 = 0.0490 vs R1 = 0.0512), but the difference is too small to conclude that inversion symmetry is broken. For the 50% Sn and 25% Sn samples, the new diffraction spots are extremely weak and rather diffuse (see Figure 1c), suggesting that ordering of the FA cations is incomplete. However, in both of these samples, a third, sharp phase transition takes place at 115 K, where the average unit cell reverts to that of the *P4/mbm* β-phase but now with an additional incommensurate modulation, as evidenced by the appearance of satellite spots with noninteger *l*-indices (Figure 1d). We refer to this as the γ′-phase in Figure 1a. The refined *q*-vectors for these samples are listed in Table 1 and in both cases are close to (0, 0, 1/6), which has previously been observed in one study of the γ-phase of FAPbI_3_^11^ but not in other reports on the same material.^10,12^ The unclear nature of γ-FAPbI_3_ has been explained in terms of considerable remaining disorder of the FA molecules, perhaps because their orientations are not fully compatible with the octahedral tilting pattern.^12,28^ Indeed, the β–γ transition in FAPbI_3_ is complex and may take place via an intermediate phase.^29^ Unfortunately, the combination of twinning with incommensurate modulation prevented us from performing full structure solutions of the γ′-phase for 50% and 25% Sn. Interestingly, aging of the samples may also influence the nature of the γ-phase; crystals of the 50% and 25% Sn compounds that had been stored for 2 years in a glovebox under a dry nitrogen atmosphere remained essentially in the β-phase when cooled to 100 K with diffraction spots corresponding to the doubled *c*-axis in the expected γ-phase barely visible and no incommensurate spots at lower temperature even though the temperature of the α–β transition was unchanged.

The established crystal structures for the mixed FAPb_1–*x*_Sn_*x*_I_3_ compounds now allow for computational studies on their electronic properties using DFT calculations. As mentioned above, one of the striking features of mixed Pb–Sn perovskites is the pronounced nonlinearity of their band gap. Here, we start by expanding the cubic unit cell of room-temperature FASnI_3_ to a 2 × 2 × 2 cubic supercell and then replace Sn by Pb to create 0%, 25%, 50%, 75%, and 100% Sn compounds, respectively. The computational details are given in the Experimental Section.

**Figure 2:**
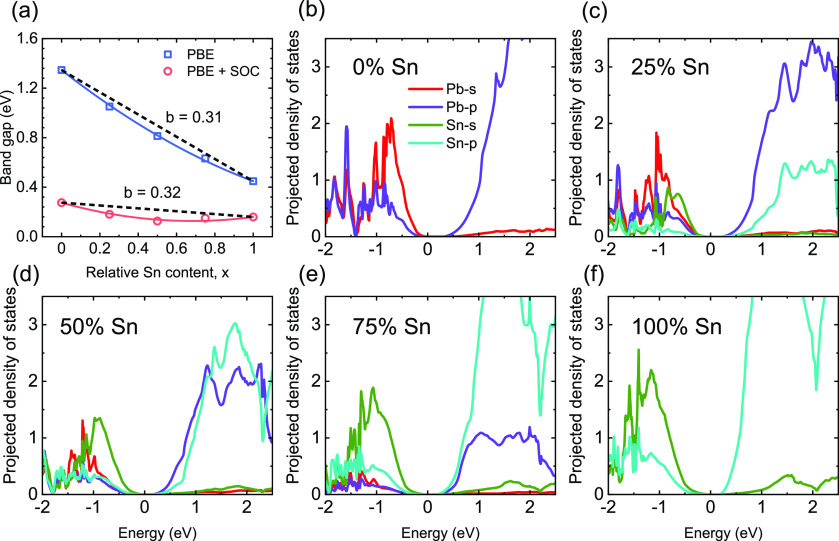
(a) Calculated band gap energies as a function of Sn content *x* using a PBE approach with and without spin–orbit coupling (SOC). (b–f) Projected density of states (PDOS) of Pb–Sn for the PBE–SOC calculations upon variation of the Sn content *x*. The valence band maximum is aligned to 0 eV.

Figure 2a shows the results for the DFT calculated band gaps as a function of relative Sn content *x*. The most favorable configurations with the lowest total energy at each discrete *x* are selected, and their corresponding atomic structures are displayed in Figure S1. The blue squares indicate the computed band gap energies using the PBE functional while the red circles indicate the calculated band gaps when the SOC is included. The band gap for the end compounds is reduced by 1.07 eV for *x* = 0 and 0.29 eV for *x* = 1 due to the inclusion of SOC. The band gap reduction in the end compounds can be also measured by considering the difference between the scalar-relativistic *p* and fully relativistic  energies: , where Δ_SOC_ is the SOC splitting energy.^30,31^ When adding a bowing term to Vegard’s law, we obtain the band gaps *E*_g_ as a continuous function of Sn content, *x*, at PBE (blue line) and PBE–SOC (red line) levels of theory: *E*_g_^PBE^(*x*) = 1.35(1 – *x*) + 0.45*x* – 0.31*x*(1 – *x*) and *E*_g_^SOC^(*x*) = 0.28(1 – *x*) + 0.16*x* – 0.32*x*(1 – *x*). The band gap shift Δ*E*_g_ owing to relativistic corrections is then expressed as Δ*E*_g_(*x*) = 1.07(1 – *x*) + 0.29*x* + 0.01*x*(1 – *x*). The first two terms are the weighted sum of the linear band gap shifts of the end compounds, which can be viewed as the composition-weighted reductions in p energy levels of Pb and Sn. The third term is the nonlinear contribution to the band gap reduction, which is negligible. This indicates that SOC has a minor effect on the band gap bowing in mixed Pb–Sn compounds.

We note that a recent solid-state NMR experiment indicated complete Pb–Sn disorder in mixed Pb–Sn perovskites without any evidence for cation segregation.^32^ This rules out any effect on the bowing by Pb–Sn short-range order as proposed by Eperon et al.^5^ However, the computational description of random alloys by periodic structures will introduce a certain degree of ordering due to the spurious correlations (“periodicity errors”). As a countermeasure, a state-of-art Special Quasirandom Structures (SQS) method has been widely used to mimic random alloys by deferring periodicity errors to more distant neighbors.^26^ Applying the SQS method to a 4 × 4 × 4 supercell in the current case, we obtain a similar bowing parameter (*b* = 0.38) as shown in Figure S2. This indicates that cation ordering, if present, can only play a minor role in band gap bowing in FAPb_1–*x*_Sn_*x*_I_3_.

To gain more insight into the origin of the band gap bowing in mixed FAPb_1–*x*_Sn_*x*_I_3_, we calculated the projected density of states (PDOS) of the five compounds upon inclusion of SOC as shown in Figure 2b–f. For ease of comparison, we only explicitly show the DOS of Pb and Sn cations for the studied compounds. The accompanying band structures and carrier effective masses are given in Figure S3 and Table S1. From the PDOS of Pb–Sn, we observe that a sizable amount of s states and a lower amount of p states of Pb–Sn contribute to the valence band maximum (VBM), while the p states of Pb–Sn provide the main contribution to the conduction band minimum (CBM). With the exception of the neat Pb-based compound, the higher atomic energy level of the Sn s states compared to Pb s states^30^ renders the VBM to be governed by the Sn–I interaction, leading to an increase in the energy of the VBM with increasing *x*. The Sn p orbital also has a slightly higher energy level than the Pb p orbital.^30^ The CBM is thus initially governed by Pb p states until a transition occurs between *x* = 0.50 and *x* = 0.75 after which the Sn p states become dominant. For higher Sn content, the contributions from the Sn p states overwhelm the Pb p states, resulting in the upshift of the CBM and consequently the increase of the band gap. We thus conclude that the nonlinearity of the band gap in mixed Pb–Sn perovskites is mainly induced by the energetic mismatch of s and p atomic levels in Pb and Sn.

**Figure 3:**
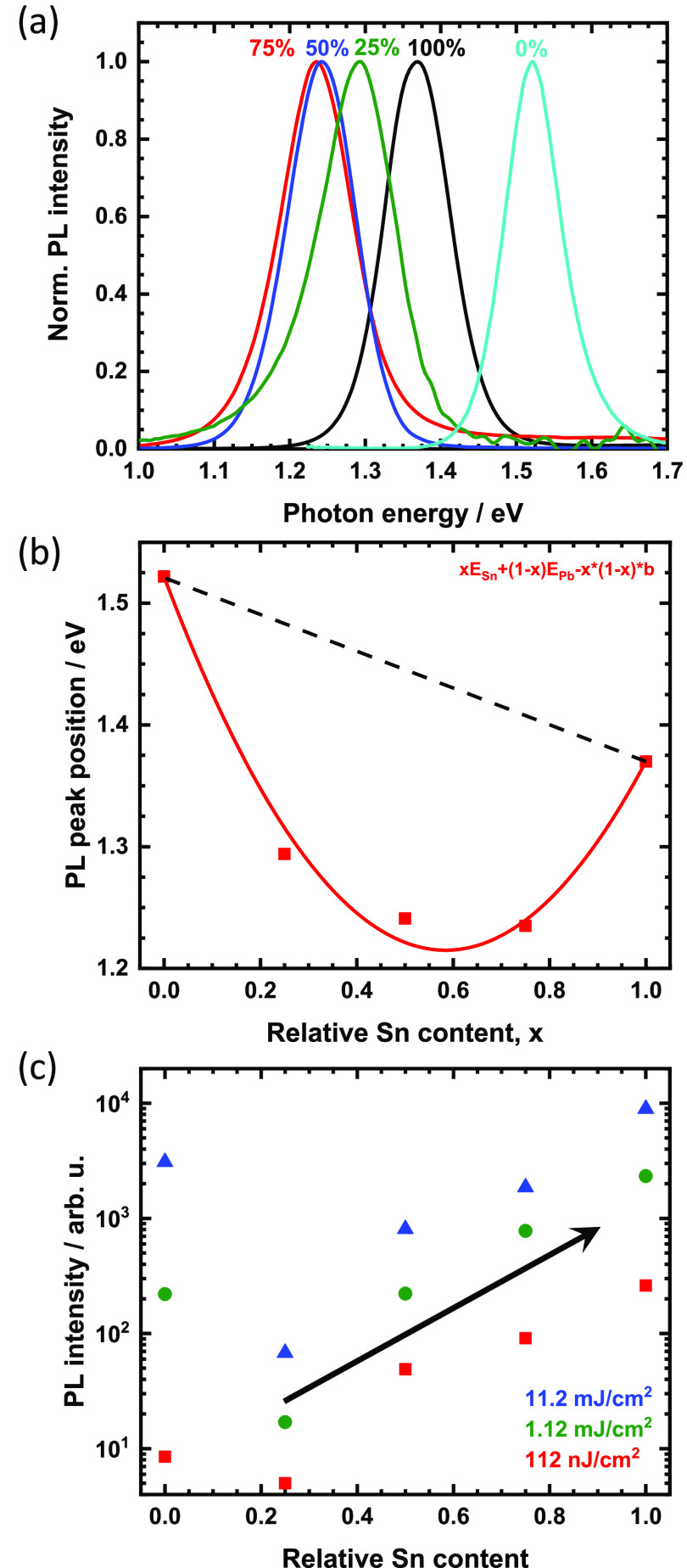
(a) Normalized photoluminescence spectra of mixed thin films with the extracted peak positions. (b) The determined bowing parameter (*b*) is 0.89. (c) The emission intensity increases upon addition of Sn (indicated by the arrow) but exhibits a relative maximum for neat FAPbI_3_.

As expected from the above-determined band gap energies, the luminescence of these compounds falls into the near-infrared spectral region. Note that our PL experiments are based on thin films as the brittle nature of the crystals did not allow for cleaving smooth surfaces and thereby prevented artifact-free measurements from being reliably performed. Thin film fabrication assured the correct and reproducible determination of the PL spectra. At room temperature, the PL peaks lie between 1.55 and 1.25 eV (800–990 nm) as shown in Figure 3a. As highlighted in Figure 3b, the nonlinear trend of the band gap also translates into a strong bowing of the extracted PL peak energies with respect to the composition. Following *E*(*x*) = *xE*_Sn_ + (1 – *x*)*E*_Pb_ – *x*(1 – *x*)*b*, a minimum energy of 1.22 eV is found around a composition of 60% Sn. Similar positions for the lowest peak energy have been found in the related systems comprising Cs and MA on the A-site.^1^ Note that although to a first approximation the PL peak position follows the band gap bowing, additional effects create a composition-dependent Stokes shift. In particular, the area of low Sn content is known to be defective and to exhibit a larger separation between PL and absorption.^33^ It is thus not surprising to find a significantly larger bowing parameter of *b* = 0.89 from the PL experiments.

Despite being known for an inferior performance in photovoltaic devices and for being prone to easy oxidation, neat tin-based compounds tend to exhibit a high luminescence intensity. Figure 3c shows how increasing the amount of lead content strongly reduces the PL intensity of mixed films. A minimum is found for 75% Pb. Neat FAPbI_3_ exhibits a similar emission intensity as compounds with low Pb content. This observation seems surprising, but bright emission of neat tin-based compounds^13,34^ and reduction upon Pb addition have been reported before.^35^ It might also be surprising in light of the longer PL lifetimes found for neat FAPbI_3_ and Pb-rich compounds.^35^ On the one hand, the intrinsic p-type doping of Sn-based samples can increase the recombination rate, leading to bright and fast emission, as long as doping is not accompanied by pronounced nonradiative channels. On the other hand, a recent report on the two neat compounds proposed that the generally broader absorption onset of tin-based compounds was responsible for a higher rate of radiative recombination.^36^ The reduction of the PLQY for mixtures is thus clearly linked to additional channels of nonradiative carrier decay. In particular, as Klug et al.^37^ and Savill et al.^33^ showed that, for the closely related Cs/FA-based variants, the mixtures with Sn content between 0.5% and 30% are particularly defective. The origin of this becomes clearer when considering the luminescence upon cooling, as presented below.

**Figure 4:**
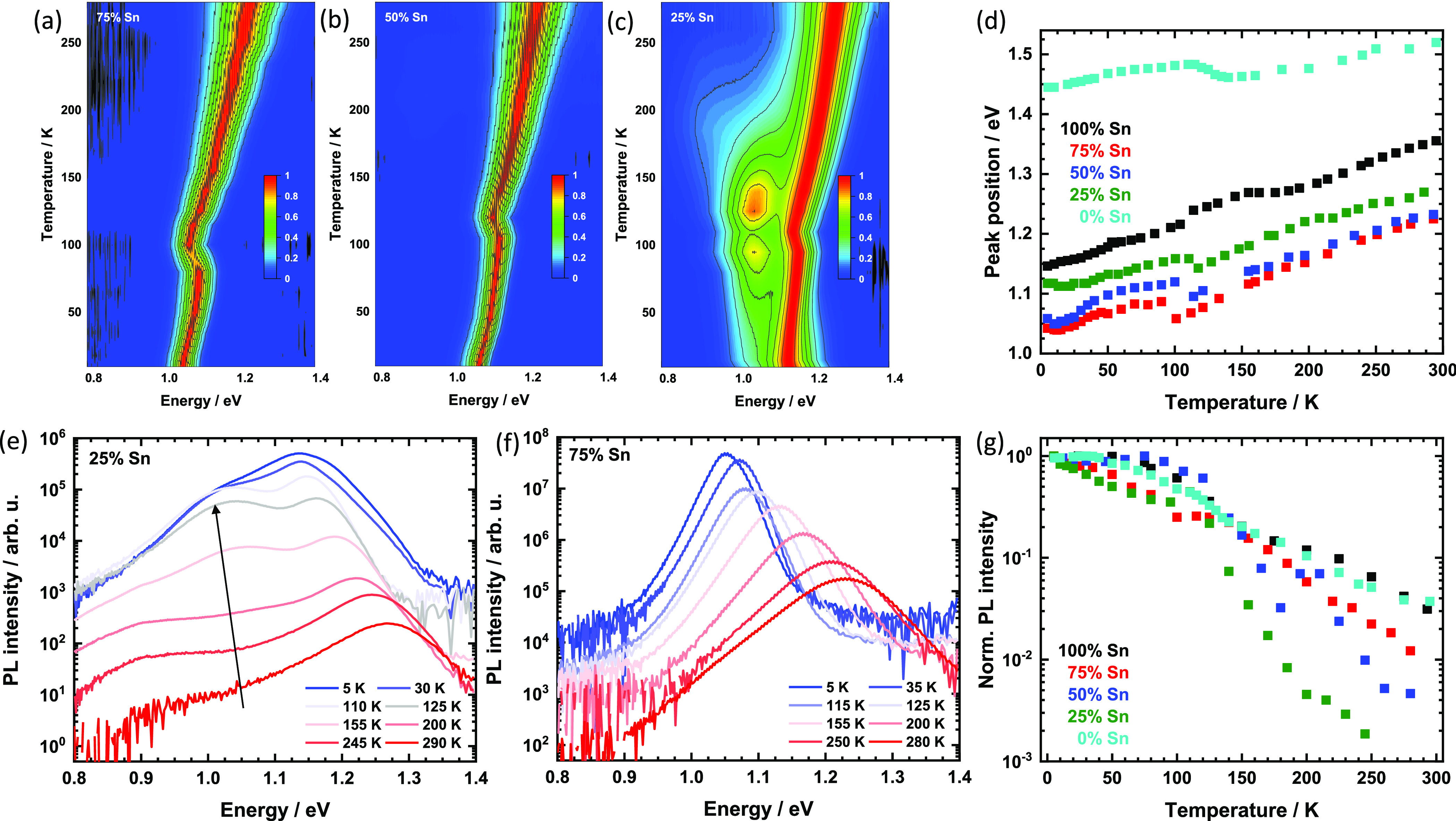
False-color plots of the normalized PL of the mixed thin films upon temperature variation (a–c) with the extracted peak energies (d). Semilogarithmic plots of PL spectra of 25% (e) and 75% (f) Sn at selected temperatures. The arrow indicates the pronounced PL band below the main peak. Temperature-dependent PL intensity of the samples normalized to the value obtained at 5.4 K (g).

Normalized steady-state spectra of the mixed compounds are given as false color plots in Figure 4a,c for a temperature range from 293 down to 5.4 K. Data on the two neat compounds have been published previously and are shown in Figure S4 for comparison.^34,38^ All compounds exhibit a pronounced PL red-shift upon cooling, which is a typical observation for metal halide perovskites.^39,40^

Similar to the case of neat FAPbI_3_, the PL line width of the 75% and 50% Sn samples is strongly reduced upon cooling (also consider Figure S5), indicating that the dominant mechanism behind the line width broadening remains the Fröhlich interaction.^38,41^ Moreover, there is also a clear discontinuity of the peak position at 100 and 110 K for 75% and 50% Sn, respectively. As exemplified by the extracted peak positions in Figure 4d, this is found around the β to γ transition at 140 K for FAPbI_3_^38,41^ but is largely absent for FASnI_3_, as discussed before.^13,34^ Although the spectra of 25% Sn are generally different, this peak shift is also observed around 115 K.

Discontinuities in the shift of the PL peak position are often good indicators for phase transitions. For the 50% and 25% Sn compounds, there is a good agreement with the transition toward the γ′-phase. However, for the 75% Sn film, the discontinuity lies right at the edge of the minimum temperature of the XRD experiments, and we did not observe a transition from γ to γ′ for this compound. The PL data thus suggest that such a phase might also exist for 75% Sn. At the same time, we note that there is some uncertainty from the fact that we considered thin films in our PL experiments, for example, because transition temperatures were previously observed to depend on the crystal grain size (in MA-based systems).^42^

Figure 4e,f shows the spectra of the 25% and 75% Sn samples in a semilogarithmic plot for selected temperatures. The curves clearly allow one to follow the band gap reduction and the overall brightening of the main peak. As expected from the normalized data in Figure 4c, pronounced PL can be observed below the main peak for 25% over a broad temperature range. Two broad, but distinct, emission bands can be identified. First, a weak band around 0.9 eV, present already at RT, becomes increasingly pronounced down to 230 K. Second, from 200 K on, a band around 1.05 eV starts to dominate the emission with an intensity on par with the main peak. Interestingly, its emission remains constant below 110 K. In contrast, the thin film containing 75% Sn in Figure 4f exhibits no such emission bands. We attribute these increasingly emissive PL bands to defect states within the band gap, underlining the defectiveness of mixed systems at low Sn concentrations.

Further insight can be obtained when considering the overall PL intensity of the main band edge emission upon temperature variation. Figure 4g shows the corresponding data normalized to the intensity at 5.4 K in a semilogarithmic plot. The two neat compounds exhibit a clear, but relatively modest, reduction of the intensity upon heating, indicating the impact of temperature-activated nonradiative decay channels. The three mixtures display a much more pronounced reduction, indicating that temperature-activated nonradiative decay is stronger in these compounds. In particular, the 25% Sn compound exhibits a drastic reduction in PL intensity around 150 K, which is the range over which the defect-related band around 1.05 eV becomes dominant.

Given the ubiquity of the strong impact of defects for low Sn contents observed here and in previous reports on related compositions,^33,35,37^ they are unlikely due to improper deposition protocols. In contrast, it is important to consider possible changes in defect chemistry occurring upon compositional variation.^19,20^ Mixed Pb–Sn compounds will likely have an intermediate behavior for the prevalence of defect types. Moreover, since the position of the band edges changes with the Sn to Pb ratio, defects that act as shallow traps can easily change their character and become deep defects in mixed compounds, leading to increased carrier recombination. Importantly, for the 25% Sn compound, the PDOS still indicates a Pb-derived CBM, which changes for high Sn content. Accordingly, our theoretical insights together with the experimentally found in-gap states suggest that the region of low Sn content is inherently susceptible to exhibiting poor optoelectronic properties due to defects. Therefore, we suggest that, where a defined band gap energy in applications based on these Pb–Sn systems is required, the corresponding compound of high Sn content should be chosen.

## Conclusions

In summary, we synthesized single crystals of mixed FAPb_1–*x*_Sn_*x*_I_3_ composition under an inert atmosphere. The crystals grew to sizes of several millimeters, and their structures were examined using single crystal XRD. At room temperature, all compounds exhibit a cubic α-phase with *Pm3̅m* symmetry akin to their neat parent compounds. Upon cooling to 100 K, all compositions undergo at least two phase transitions to a tetragonal β-phase of *P4/mbm* symmetry above 250 K and a second tetragonal γ-phase around 155 K and below. For 25% and 50% Sn content, crystallographic studies furthermore reveal an incommensurate structure we term γ′ in which an unresolvable superstructure is observed.

To ensure their accuracy, the so-obtained structures provide the basis for DFT calculations. We identify a strong impact of spin–orbit coupling on the absolute band gap energy, whereas the actual bowing is predominantly determined by the fundamental differences in the s and p atomic orbital energies of Pb and Sn. For a broad range of compositions, the VBM is determined by Sn s states and the CBM, by Pb p, resulting in a narrower band gap than either of the neat compounds possess.

PL spectra offer a similar nonlinear trend of the peak energy, albeit with a more pronounced bowing, due to a composition-dependent Stokes shift. Mixed compounds with low Sn content are particularly strongly governed by defect states in the band gap, leading to bright luminescence at low energy upon temperature reduction. The changes in character and position of the VBM and CBM are therefore identified to result in a composition-dependent defect chemistry with a strong impact on the recombination of charge carriers.

Refuting the general idea in the perovskite community that “lead means stable and tin means unstable”, this work suggests that mixed Pb–Sn iodide perovskites are inherently defective at low Sn concentrations. On the contrary, applications that require a band gap energy covered by these mixtures should be based on compounds with a high Sn content.

## Supporting Information Available

Atomic structure employed in the DFT calculations, band gap bowing according to the SQS method, band structure and effective masses, and temperature-dependent PL spectra of the neat compounds and corresponding line widths (PDF)
Crystallographic information files for the mixed compounds: FAPb_0.75_Sn_0.25_I_3_, 295 K, *Pm3m*; FAPb_0.75_Sn_0.25_I_3_, 200 K, *P4/mbm*; FAPb_0.5_Sn_0.5_I_3_, 295 K, *Pm3m*; FAPb_0.5_Sn_0.5_I_3_, 200 K, *P4/mbm*; FAPb_0.25_Sn_0.75_I_3_, 295 K, *Pm3m*; FAPb_0.25_Sn_0.75_I_3_, 200 K, *P4/mbm*; FAPb_0.25_Sn_0.75_I_3_, 100 K, *P4/mbm* (ZIP)
