# Supplementary material for: Compositional Variation in FAPb1–xSnxI3 and Its Impact on the Electronic Structure: A Combined Density Functional Theory and Experimental Study
Source: ACS Appl Mater Interfaces. 2022 May 5;14(30):34253–61. doi: 10.1021/acsami.2c00889 (PMC9353781; doi:10.1021/acsami.2c00889)
Supplement: Supplementary file 1 — am2c00889_si_001.pdf [file am2c00889_si_001.pdf]

Supporting Information for: Compositional Variation in $FAPb_{1-x}Sn_xI_3$ and its Impact on the Electronic Structure: A Combined DFT and Experimental Study

Simon Kahmann,[†,‡,△] Zehua Chen,[¶,§,△] Oleh Hordiichuk,[‖,⊥] Olga Nazarenko,[‖,⊥] Shuyan Shao,[†,#] Maksym V. Kovalenko,[‖,⊥] Graeme R. Blake,[@] Shuxia Tao,[*,¶,§] and Maria A. Loi[*,†]

†*Photophysics and OptoElectronics Group, Zernike Institute of Advanced Materials, University of Groningen, Nijenborgh 4 NL-9747 AG, Groningen, The Netherlands*

‡*Current address: Cavendish Laboratory, University of Cambridge, JJ Thomson Avenue, CB30HE Cambridge, United Kingdom*

¶*Materials Simulation and Modelling, Department of Applied Physics, Eindhoven University of Technology, 5600 MB Eindhoven, The Netherlands*

§*Center for Computational Energy Research, Department of Applied Physics, Eindhoven University of Technology, Eindhoven 5600 MB, The Netherlands*

‖*Department of Chemistry and Applied Biosciences, ETH Zürich, Vladimir Prelog Weg 1, Zürich, CH-8093, Switzerland.*

⊥*EMPA-Swiss Federal Laboratories for Materials Science and Technology, Überlandstraße 129, Dübendorf, CH-8600, Switzerland*

#*Current address: Institute of Molecular Aggregation Science, Tianjin University, Tianjin 300072, China*

@*Solid State Materials for Electronics, Zernike Institute of Advanced Materials, University of Groningen, Nijenborgh 4 NL-9747 AG, Groningen, The Netherlands*

△*These authors contributed equally.*

E-mail: s.x.tao@tue.nl; m.a.loi@rug.nl

ADDITIONAL DATA

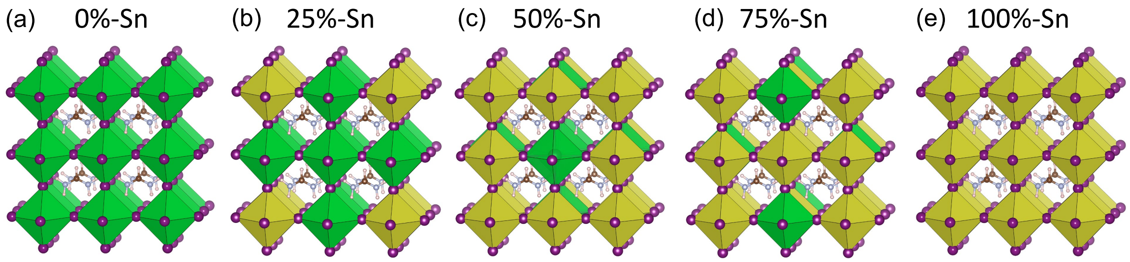

Figure S1: Atomic structures for the favourable compounds with different Sn contents at $x = 0.0$, 0.25, 0.50, 0.75, and 1 (a-e). Purple spheres: I. Spheres inside green (yellow) octahedra: Pb (Sn). Cationic molecules in between octahedra: formamidinium (FA).

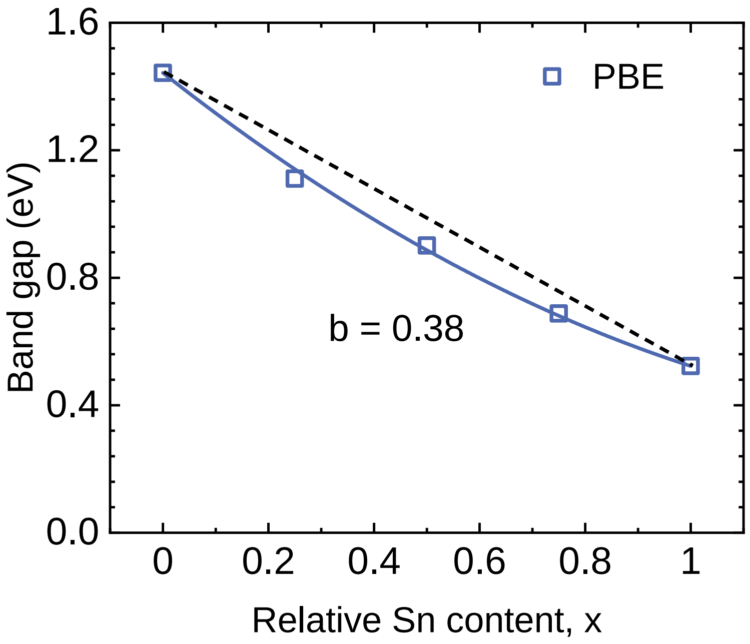

Figure S2: Band gaps calculated using the Special Quasirandom Structures (SQS) method. Here the $4 \times 4 \times 4$ supercell containing 64 formula units is used. To reduce the computational cost, the SOC is not included since it has negligible impact on band gap bowing.

Table S1 shows the effective masses for the five compounds, as extracted from the band dispersions as shown in Figure S3. The effective mass of electrons and holes at low concentrations is inversely proportional to the parabolic curvature of the band dispersion that close to the VBM and CBM. The analytical expression of effective mass is thus given by

$m^* = \hbar^2 \left[\frac{\partial^2 E(k)}{\partial^2 k}\right]^{-1}$, where $\hbar$ is the reduced Planck constant and $E(k)$ is the energy dispersion described by band structures along the vector k in reciprocal space. The impact of the composition on the effective masses is relatively minor.

Table S1: The effective masses of the electrons and holes (in unit of the electron static mass m_0) for the five compounds upon the inclusion of SOC.

	0% Sn	25% Sn	50% Sn	75% Sn	100% Sn
Electron	R → M: 0.071	G → R: 0.062	G → R: 0.061	G → R: 0.056	R → M: 0.097
	R → G: 0.066	G → X: 0.069	G → X: 0.075	G → X: 0.081	R → G: 0.063
Hole	R → M: 0.076	G → R: 0.070	G → R: 0.056	G → R: 0.048	R → M: 0.055
	R → G: 0.079	G → X: 0.069	G → X: 0.070	G → X: 0.064	R → G: 0.045

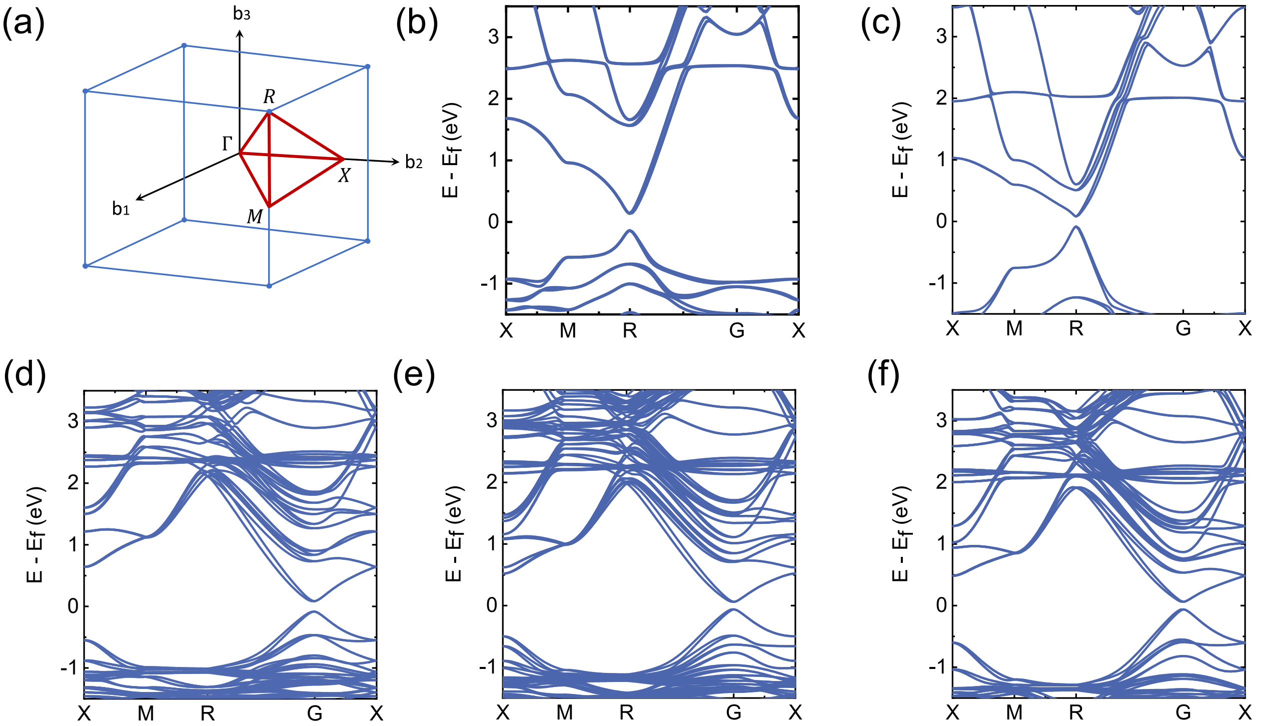

Figure S3: (a) High symmetry points within the Brillouin zone of cubic perovksite structures. (b)-(c) The band structures for pure $FAPbI_3$ and $FASnI_3$, for which we have used cubic unit cells. (d)-(f) The band structures for the compounds with Sn content $x = 0.25$, 0.50 and 0.75, respectively. For these three compounds, we have used $2\times2\times2$ cubic supercells. For all band structure calculations in this work we have taken the spin-orbit coupling into account.

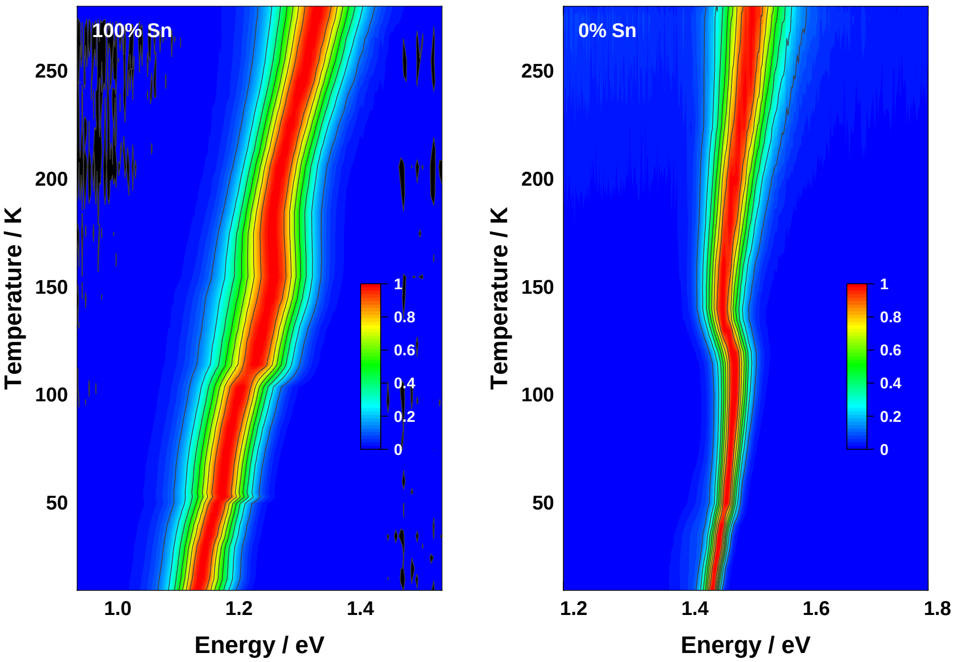

Figure S4: Temperature-dependent PL of the two neat compounds based on previous publications.[1,2] The false colour plots of $FASnI_3$ (a) and $FAPbI_3$ (b) highlight the different impact of phase transitions on the PL peak position as well as stark differences in the linewidth narrowing upon cooling.

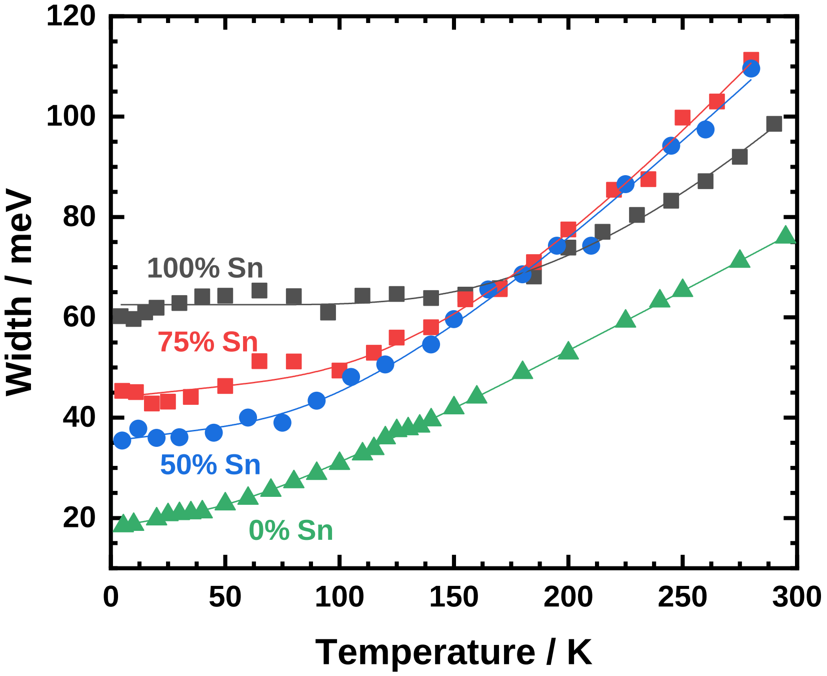

Figure S5: Temperature dependence of the PL linewidth of the different compounds. The 25% Sn sample was excluded due to the strong impact of the radiative defects. Similar to neat $FAPbI_3$, the mixed compounds exhibit a pronounced increase of the PL linewidth over a broad temperature range, but with a larger residual width at low temperature. This indicates a larger inhomogeneous broadening.

References

(1) Fang, H.-H.; Wang, F.; Adjokatse, S.; Zhao, N.; Even, J.; Antonietta Loi, M. Photoexcitation dynamics in solution-processed formamidinium lead iodide perovskite thin films for solar cell applications. *Light Sci. Appl.* **2016**, *5*, e16056.

(2) Kahmann, S.; Shao, S.; Loi, M. A. Cooling, Scattering, and Recombination—The Role of the Material Quality for the Physics of Tin Halide Perovskites. *Adv. Funct. Mater.* **2019**, *29*, 1902963.
